# Excision of left atrial myxoma under perfused ventricular fibrillation with hypothermia after coronary artery bypass grafting

**DOI:** 10.1186/s13019-023-02400-4

**Published:** 2023-10-10

**Authors:** Shaobo Pan, Yalin Wang, Yanjia Gu, Weidong Li, Hongfei Xu

**Affiliations:** 1 Department of Operation Room, School of Medicine, The First Affiliated Hospital of Zhejiang University, Hangzhou, China; 2 Department of Cardiovascular Surgery, School of Medicine, The First Affiliated Hospital of Zhejiang University, Number 79 Qingchun Road, Hangzhou, China

**Keywords:** Myxoma, Redo surgery, Ventricular fibrillation, Coronary artery bypass grafting

## Abstract

**Background:**

Redo heart surgery has become increasingly common but involves additional high surgical risk, especially redo surgery after coronary artery bypass grafting (CABG).

**Case presentation:**

In this study, we report the case of a 57-year-old Chinese male with left atrium myxoma who had previously undergone CABG. Common surgical methods usually include aortic cross-clamping, administering cold cardioplegia perfusion to protect the myocardium, opening the heart, and then removing the tumor. However, for patients with previous CABG, redo thoracotomy and ascending aortic cross-clamping present a greater risk of damage to the grafted vessels. In this study, we chose a right lateral mini-thoracotomy incision and hypothermia-induced ventricular fibrillation to minimize damage and avoid any adverse effects on the bridge vasculature. The patient recovered uneventfully and was discharged seven days after surgery.

**Conclusions:**

For patients with previous CABG, minimally invasive right thoracotomy under perfused ventricular fibrillation with hypothermia is safe and reliable and can prevent potential damage to the ascending aorta and graft.

## Background

Cardiac myxoma (CM) is the most common type of primary cardiac tumor, accounting for 58.14% of all cardiac tumors [1]. CM can occur in all chambers of the heart but arises most commonly in the left atrium (60–80%), interatrial septum, and fossa ovalis [2]. Embolic manifestations are one of the most common symptoms of CM and are associated with high morbidity and mortality. The emboli can involve cerebral or other arteries depending on the region where the CM arises. Common surgical methods usually include aortic cross-clamping, administering cold cardioplegia perfusion to protect the myocardium, opening the heart, and then removing the tumor. However, for patients who have previously undergone coronary artery bypass grafting (CABG), secondary thoracotomy, ascending aortic cross-clamping, and Y-shaped tube cannulation present a greater risk of damage to grafted vessels.

### Case presentation

**Fig. 1:**
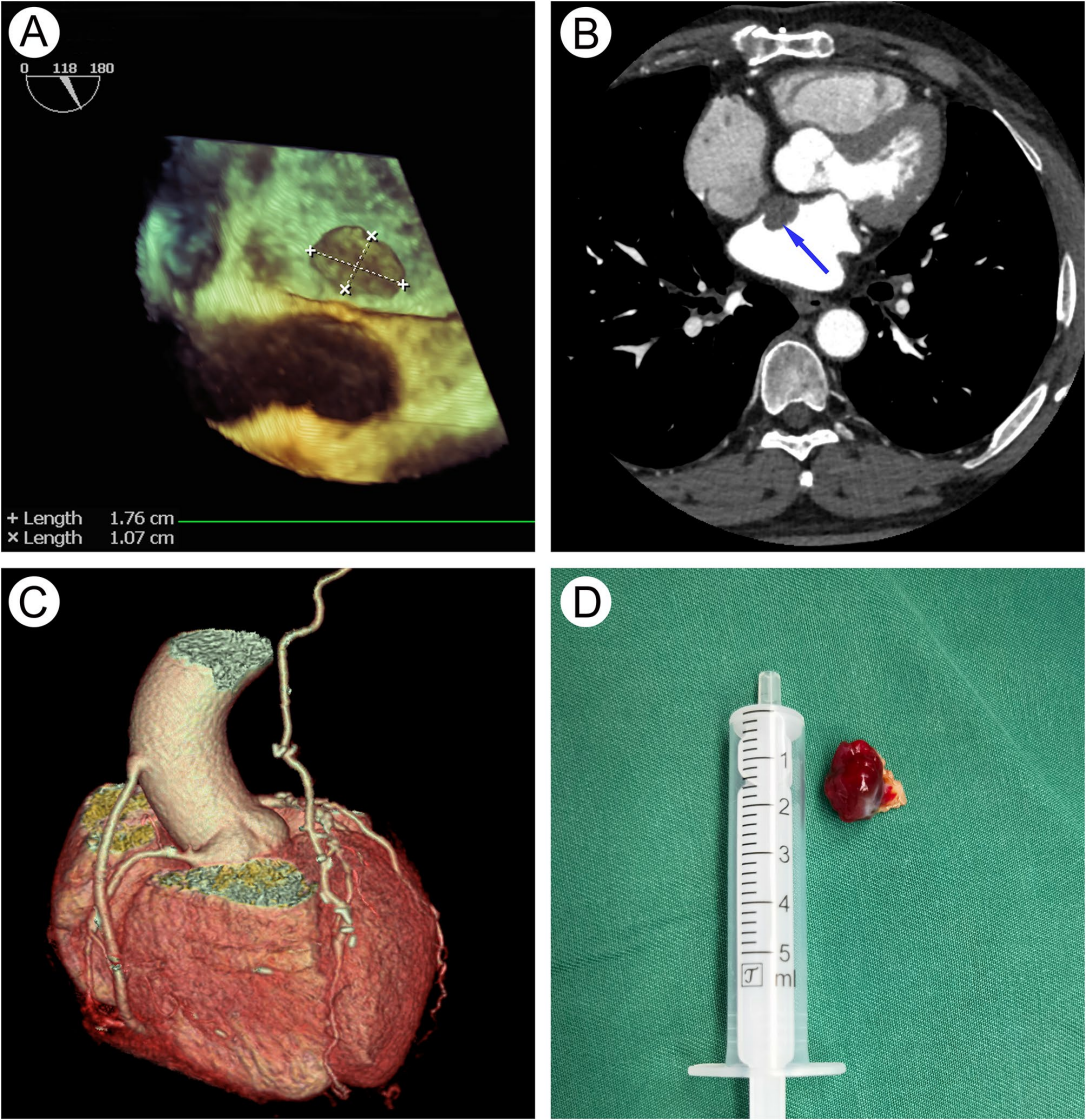
Excision of left atrial myxoma after coronary artery bypass grafting. **a** 3D transesophageal echocardiography revealed a hyperechoic lesion (1.76 cm × 1.07 cm) attached to the interatrial septum in the left atrium. **b** Computed tomography coronary angiography confirmed the mass with a low-density shadow. **c** The coronary artery bypass grafts of the saphenous vein graft and left internal mammary artery graft were smooth. **d** A pedunculated, jelly-like cardiac myxoma was completely resected

A 57-year-old Chinese male who underwent CABG five years ago was recently admitted because of a left atrial mass detected by ultrasonic cardiogram at another institution. After anticoagulant therapy for one year, the mass did not change. Except for occasional chest stuffiness after an activity, he felt no obvious discomfort. At our center, transesophageal echocardiography revealed a hyperechoic lesion attached to the interatrial septum in the left atrium measuring approximately 1.8*1.1 cm (Fig. 1a). No obvious abnormalities were observed in the rest of the cardiac structures, and the aortic valve opened and closed properly. Computed tomography coronary angiography confirmed the mass (Fig. 1b) and showed that the coronary artery bypass grafts were smooth without any evidence of stenosis or occlusion (Fig. 1c). The patient underwent reoperation through a right lateral mini-thoracotomy incision (6 cm) at the fourth intercostal space. An endoscope was inserted via a separate anterior axillary incision at the third intercostal space for video assistance. Dense pericardial adhesions were observed, and the ascending aorta could not be fully exposed. Body temperature was lowered to 28 ºC to induce ventricular fibrillation under the support of cardiopulmonary bypass instituted through femoro-femoral cannulation without the infusion of cardioplegic solution (perfusion pressure was maintained as high as possible, preferably above 60 mmHg). A 17 F femoral artery cannula (MAQUET) and 23 F femoral vein cannula (MAQUET) were used according to standard procedure, and the tip of the femoral vein cannula was placed near the superior vena cava. The left atrium was opened through the interatrial groove approach without aortic cross-clamping. After a left atrial retractor was implanted, we placed a drainage tube across the mitral valve to the left ventricle, and the tip of another suction tube was placed near the ostium of the left superior pulmonary vein. In addition, we continuously filled the surgical field with carbon dioxide gas, and observed that the tumor was hanging on the atrial septum, pedunculated and jelly-like, and was completely resected with the atrial septum (Fig. 1d). The interatrial groove was then closed with a running suture in two layers. After the temperature was restored and the air in the left ventricle was completely evacuated through a left ventricle drainage tube (monitored by real-time esophageal echocardiography), the heart automatically resumed beating, and the patient was successfully weaned from extracorporeal circulation. A chest drainage tube was used for drainage and pulmonary re-expansion. The total fibrillatory arrest time was 35 min, and the cardiopulmonary bypass time was 65 min. Histopathology examination confirmed the diagnosis of myxoma. The patient woke up two hours after the operation. The drainage volume was 400 ml on the first day and 100 ml on the second day. The drainage tube was removed on the third day. The intensive care unit (ICU) time was 36 h. The patient recovered uneventfully and was discharged seven days after surgery.

## Discussion

When a myxoma is diagnosed, the treatment of choice is surgical excision [3]. During conventional surgery, cardiopulmonary bypass and aortic cross-clamping are established in all patients. Median sternotomy with distal ascending aortic and bicaval cannulation is the most common approach used during CM resection [4].

Since minimally invasive approaches have become the standard treatment in isolated valvular surgery in the last few years, several groups have also applied this approach in the excision of intracardiac tumors. Lee HP et al. [5] compared the outcomes of their right mini-thoracotomy approach versus the median sternotomy approach and showed that the minimally invasive right mini-thoracotomy approach was associated with a lower incidence of postoperative complications and a shorter postoperative recovery period. Bianchi G et al. [6] reported their outcomes of video-assisted minimally invasive cardiac myxoma resection and posited that it is an effective and reproducible strategy in all patients requiring expedited surgery for left atrial myxoma.

In our center, valvular surgery through minimally invasive approaches is widely used, which allowed us to try this approach in an atrial myxoma excision. Surgery on the left heart is routinely performed through an interatrial groove incision (left atriotomy) rather than a right atrial approach. The main reasons are as follows. First, this method does not require dissociation and ligation of the superior vena cava and inferior vena cava, which can simplify the operation steps. Second, it does not require internal jugular vein or superior vena cava cannulation, which shortens the operation time and decreases the risk. Third, the incision and suture of the atrial septum may cause difficulties in future percutaneous interventional operations, specifically atrial septal puncture. Fourth, for this kind of left atrial myxoma surgery, the overall shape and pedicle position of the tumor can be seen through the atrial septal incision directly, avoiding the potential risk of tumor rupture or falling caused by atrial septal incision. In some special cases, such as severe atrial septal adhesion and a huge right atrium, we will also use an atrial septal incision to complete the operation. In this case, the patient had undergone CABG via median sternotomy five years prior. Reoperation through median sternotomy may cause difficulty in exposing the ascending aorta because of adhesions, leading to an increased risk of bleeding. Therefore, we preferred a minimally invasive approach over routine median sternotomy to avoid extensive dissections.

In addition, the operation five years ago used a left internal mammary artery graft to a left anterior descending and saphenous vein graft to the right coronary artery. This arrangement would make any procedure on the ascending aorta and prone to causing adverse effects on the proximal saphenous vein graft, including aortic cross-clamping and insertion of the Y tube at the aortic root [7], which are necessary for routine minimally invasive surgery. To avoid these problems, studies have tried to perform reoperative minimally invasive mitral valve surgery through a right mini-thoracotomy on patients with previous coronary artery bypass grafting, and the results confirmed that minimally invasive right thoracotomy without aortic cross-clamping is an excellent alternative to conventional redo sternotomy for reoperative left heart surgery [8, 9]. In our case, we utilized hypothermic ventricular fibrillation arrest. Thus, no action was taken to the ascending aorta, and the risk of damaging the saphenous vein graft was minimized. It should be noted that this method is not recommended for patients with moderate or severe aortic valve regurgitation or those with a previous history of mechanical aortic valve replacement, because there will be a large amount of blood regurgitation in the left ventricle, causing cardiac distension or seriously affecting the surgical field of view. One-year follow-up showed that the patient recovered uneventfully.

## Conclusions

In summary, we reported a case of left atrial myxoma in a patient with previous CABG. We chose a right lateral mini-thoracotomy incision and hypothermia-induced fibrillation arrest to avoid damaging the saphenous vein graft. The patient recovered uneventfully after surgery, which showed that this procedure was the correct choice. For patients with previous CABG, minimally invasive right thoracotomy under perfused ventricular fibrillation with hypothermia is safe and reliable and can prevent potential damage to the ascending aorta. We look forward to data from a larger sample in the future to support our view.

## Data Availability

Not applicable.

## Abbreviations

CM: cardiac myxoma
CABG: coronary artery bypass grafting
